# A High-Fat, High-Fructose Diet Induces Antioxidant Imbalance and Increases the Risk and Progression of Nonalcoholic Fatty Liver Disease in Mice

**DOI:** 10.1155/2016/5029414

**Published:** 2016-02-25

**Authors:** Kanokwan Jarukamjorn, Nattharat Jearapong, Charinya Pimson, Waranya Chatuphonprasert

**Affiliations:** ^1^Research Group for Pharmaceutical Activities of Natural Products Using Pharmaceutical Biotechnology (PANPB), Faculty of Pharmaceutical Sciences, Khon Kaen University, Khon Kaen 40002, Thailand; ^2^Faculty of Medicine, Mahasarakham University, Mahasarakham 44000, Thailand

## Abstract

Excessive fat liver is an important manifestation of nonalcoholic fatty liver disease (NAFLD), associated with obesity, insulin resistance, and oxidative stress. In the present study, the effects of a high-fat, high-fructose diet (HFFD) on mRNA levels and activities of the antioxidant enzymes, including superoxide dismutase (SOD), catalase (CAT), and glutathione peroxidase (GPx), were determined in mouse livers and brains. The histomorphology of the livers was examined and the state of nonenzymatic reducing system was evaluated by measuring the glutathione system and the lipid peroxidation. Histopathology of the liver showed that fat accumulation and inflammation depended on the period of the HFFD-consumption. The levels of mRNA and enzymatic activities of SOD, CAT, and GPx were raised, followed by the increases in malondialdehyde levels in livers and brains of the HFFD mice. The oxidized GSSG content was increased while the total GSH and the reduced GSH were decreased, resulting in the increase in the GSH/GSSG ratio in both livers and brains of the HFFD mice. These observations suggested that liver damage and oxidative stress in the significant organs were generated by continuous HFFD-consumption. Imbalance of antioxidant condition induced by long-term HFFD-consumption might increase the risk and progression of NAFLD.

## 1. Introduction

Nonalcoholic fatty liver disease (NAFLD) is a condition in which excessive fat accumulates in the liver of a patient who drinks little or no alcohol. The development of NAFLD is associated with obesity, insulin resistance (diabetes mellitus type 2), and hyperlipidemia [1] and is characterized by the accumulation of fatty acids, especially excess triglycerides, in hepatocytes [2]. NAFLD may also be associated with elevated liver enzyme levels, abnormal liver function, and disruption of the metabolism, secretion, and excretion of lipids [3]. Hepatic steatosis (more than 5.5% hepatic triglyceride content) is the first stage of NAFLD and is followed by liver injury in the next stage of the disease, called nonalcoholic steatohepatitis (NASH) [4]. Cytokine production is altered in the NASH stage which is further characterized by lobular inflammation, hepatocyte ballooning, and fibrosis [5]. Other factors, such as oxidative injury or oxidative stress, are required to shift from NASH to liver cirrhosis, before finally leading to hepatocellular carcinoma, in severe cases [6]. Oxidative stress and lipid peroxidation are crucial pathophysiological mechanisms in NAFLD [7]. Oxidative stress can be detected by measuring the activity of antioxidative enzymes, for example, superoxide dismutase (SOD), catalase (CAT), and glutathione peroxidase (GPx), as well as the level of malondialdehyde (MDA), particularly in the liver. Increases in SOD and GPx activity and SOD, GPx, and CAT mRNA expression in the livers of rabbits fed a high-fat diet have indicated a significant role for the antioxidant system in NAFLD [8].

The increasing prevalence of obesity, metabolic syndrome, and NAFLD has been connected to excess caloric intake, such as the excessive consumption of processed food with high fructose levels [9]. High fructose consumption has been implicated in the progression and severity of NAFLD by promoting* de novo* lipogenesis and increasing insulin resistance, oxidative stress, inflammation, and fibrosis [10, 11]; however the mechanism responsible is still unknown [12].

In the current study, we included fructose and transfats in a high-energy diet to induce experimental NAFLD in mice [10, 13]. Here, we describe the effect of this high-fat, high-fructose diet (HFFD) on the expression and activity of the SOD, CAT, and GPx antioxidant enzymes and the overall oxidative state in terms of total glutathione, reduced glutathione (GSH), and oxidized glutathione (GSSG). This study aims to elucidate the relationship between high-fat, high-fructose consumption, the oxidant-antioxidant balance, and the risk and progression of NAFLD.

## 2. Materials and Methods

### 2.1. Chemicals

Fructose syrup (55%) was purchased from Chao Khun Agro Products Co., Ltd. (Bangkok, Thailand). Bovine serum albumin (BSA), 5,5-dithiobis-(2-nitrobenzoic acid) (DTNB), glutathione reductase, oxidized glutathione (GSSG), reduced glutathione (GSH), nitrotetrazolium blue chloride (NBT), reduced *β*-nicotinamide adenine dinucleotide phosphate (NADPH), standard malondialdehyde (MDA), standard superoxide dismutase (SOD) from bovine erythrocytes, 4-vinylpyridine (4-VP), and xanthine oxidase were from Sigma Aldrich (St. Louis, MO, USA). Hydrogen peroxide (H_2_O_2_) was obtained from Fisher Scientific (Leicestershire, UK). 2-Thiobarbituric acid (TBA) was supplied by Fluka Chemika Co. (Steinheim, Switzerland). Trizol® was supplied by Invitrogen® (Carlsbad, CA, USA). ReverTraAce® and* Taq* DNA polymerase were purchased from Toyobo Co., Ltd. (Osaka, Japan) and Vivantis Technologies Sdn. Bhd. (Selangor Darul Ehsan, Malaysia), respectively. The random primers and RNase inhibitor were products of Takara Bio Inc. (Shiga, Japan). The forward and reverse primers for CAT, CuZn-SOD, Mn-SOD, GPx, and GAPDH (Table 1) were synthesized by Bio Basic, Inc. (Markham, Ontario, Canada). All other laboratory chemicals were of the highest available purity from commercial suppliers.

### 2.2. Animal Treatments

Seven-week-old male ICR mice were obtained from the National Laboratory Animal Center (Mahidol University, Thailand). All mice were housed on wood chip bedding in polysulfone cages with water and commercial regular diet (RD, SmartHeart® from Perfect Companion Pet Care Company, Thailand; 24% crude protein, 4.5% crude fat, 5% fiber, 10% moisture, 10% ash, 1% calcium, 0.7% phosphorous, and vitamins A, D3, and E) supplied* ad libitum* in the Northeast Laboratory Animal Center, Khon Kaen University, Thailand, and acclimated for a week before dosing under the supervision of the Animal Ethics Committee for Use and Care of Khon Kaen University (approval number AEKKU 92/2555). The mice quarters were air conditioned (23 ± 2°C) and controlled for humidity 45 ± 2% RH and had a 12 h light/dark cycle.

The high-fat, high-fructose diet (HFFD) mice were additionally given hydrogenated soybean oil intragastrically (1 mL/day), which consisted of 44.1% (w/w) saturated fat and 0.2% (w/w) trans fatty acid (certified by Institute of Nutrition, Mahidol University, Thailand), with the addition of 20% (w/v) fructose in their drinking water supplied* ad libitum* for 2, 4, and 8 weeks (*n* = 5 for each group). The control mice (*n* = 5) were fed with commercial regular diet (RD) and water* ad libitum* for 4 weeks. The ages of all of the mice at the end of treatment were between 10 and 16 weeks, consistent with adult-aged mice (2–4 months). Levels of antioxidant enzymes and lipid peroxidation, including the GSH/GSSG ratio, have been proposed to be equivalent in the same age-range, for example, young (4–6 weeks), adult (3 months), middle-aged (9 months), and aged (18–24 months) [14]. All mice were sacrificed 24 h after the last treatment, and their organs (livers and brains) were immediately excised and either used for the isolation of total RNA or immediately stored at −80°C for further analysis.

### 2.3. Tissue Fixation, Processing, and Staining

The liver tissue was prepared for histomorphological examination according to the method of Jearapong et al. [15] with some modifications. Briefly, a fragment of liver tissue was washed immediately in phosphate buffered saline then fixed by immersion in 10% neutral-buffered-formalin overnight and dehydrated for paraffin embedding. The paraffin piece was cut into 5 mm sections using a microtome (Microm HM315, Thermo Scientific, Walldorf, Germany) and placed on a microscope slide. The embedded tissue was stained with hematoxylin and eosin (H&E) and evaluated for histomorphological features at 200x magnification using an Axiostar Plus microscope (Carl Ziess, Oberkochen, Germany).

### 2.4. Semiquantification of CAT, SOD, and GPx mRNA Levels

Mouse CAT, CuZn-SOD, Mn-SOD, GPx, and glyceraldehyde 3-phosphate dehydrogenase (GAPDH) mRNAs were semiquantified using RT-PCR. Total RNA was reverse-transcribed using ReverTraAce (Toyobo Co., Ltd.) [16] and the cDNA was amplified with specific primers under the conditions recommended by the supplier, Vivantis Technologies Sdn. Bhd. (Selangor Darul Ehsan, Malaysia). The PCR program conditions were set according to the methods of Chatuphonprasert et al. [17], Lao-Ong et al. [18], and Kondo et al. [19] with some modifications (Table 2). The PCR products were separated using 1.5% agarose gel electrophoresis and detected under ultraviolet (UV) light in the presence of Novel Juice from GeneDirex® (Bio-Helix Co., Ltd., Taiwan). The target PCR products were normalized to GAPDH and semiquantified using a Syngene® gel documentation machine (Ingenius L, Cambridge, UK) and the GeneTools match program (Syngene).

### 2.5. Determination of SOD Activity

The level of SOD activity in liver and brain homogenates was determined by the inhibition of formazan formation [17]. A chloroform and ethanol extract of liver or brain homogenate was mixed with the reagent mixture of xanthine, ethylenediamine tetraacetic acid (EDTA), NBT, Na_2_CO_3_, and BSA, followed by xanthine oxidase. The reaction was incubated at 25°C for 20 min and terminated with CuCl_2_. The absorbance of formazan was measured at a wavelength of 550 nm. The percentage of formazan inhibition was compared with a bovine CuZn-SOD standard.

### 2.6. Determination of CAT Activity

The level of CAT activity was assessed according to the method of Chatuphonprasert et al. [17]. Briefly, sample homogenates were incubated in a H_2_O_2_ substrate at 37°C for 1 min before the addition of ammonium molybdate. The absorbance of the resulting yellow complex was measured at a wavelength of 405 nm. CAT activity is measured as the percentage inhibition of complex formation compared with a bovine hepatic CAT standard.

### 2.7. Determination of GPx Activity

The level of GPx activity was determined according to the method of Chatuphonprasert et al. [17]. The reaction mixture, consisting of sample homogenate, sodium phosphate buffer (pH 7.4), EDTA, and sodium azide, was incubated at room temperature for 10 min followed by the addition of the GSH substrate. The reaction was initiated by adding H_2_O_2_ and terminated using 5-sulfosalicylic acid (SSA). The reaction mixture was then centrifuged at 1,900 rpm for 15 min, and the GSSG content of the supernatant was used to assess GPx activity. The results are expressed as unit/mg protein, where a unit of GPx activity was defined as mmol of GSSG formed/min at 30°C and pH 7.4 [19].

### 2.8. Assessment of the Total Glutathione Content and the GSH to GSSG Ratio

Glutathione content was determined according to the method of Chatuphonprasert et al. [17]. The sample homogenate was deproteinized with SSA and kept at 2–8°C for 10 min before being centrifuged at 10,000 ×g at 4°C for 10 min. For the determination of total glutathione, the supernatant was mixed with the reaction mixture, which consisted of EDTA, NADPH, DTNB, and glutathione reductase in PBS buffer (pH 7.0). The absorbance of the thiol anions at a wavelength of 405 nm was measured every 60 s for 5 min using a spectrophotometer. To determine the GSSG content, the homogenate was treated with 4-VP before the addition of the reaction mixture and then incubated at room temperature for 1 h. Total glutathione, GSH, and GSSG contents were determined by comparing the net slope of the samples with the slope of the standard curve of GSH or GSSG [17].

### 2.9. Determination of Lipid Peroxidation

The thiobarbituric acid (TBA) assay was used to determine lipid peroxidation according to the method of Chatuphonprasert et al. [17] with a few modifications. The sample homogenates or MDA standards were incubated at 37°C for 1 h before addition of trichloroacetic acid, TBA, and acetic acid. The reaction mixtures were then boiled for 15 min, and the TBA-reactive species (TBARS) were quantified using a spectrofluorometer at an emission wavelength of 551 nm and an excitation wavelength of 528 nm.

### 2.10. Statistical Analysis

The results were analyzed using a one-way analysis of variance (ANOVA) followed by Tukey's* post hoc* test (version 17; SPSS Inc., Chicago, IL, USA). *p* < 0.05 was considered to be statistically significant.

## 3. Results

### 3.1. Histomorphological Features of the Livers of Mice Fed a High-Fat, High-Fructose Diet

**Figure 1:**
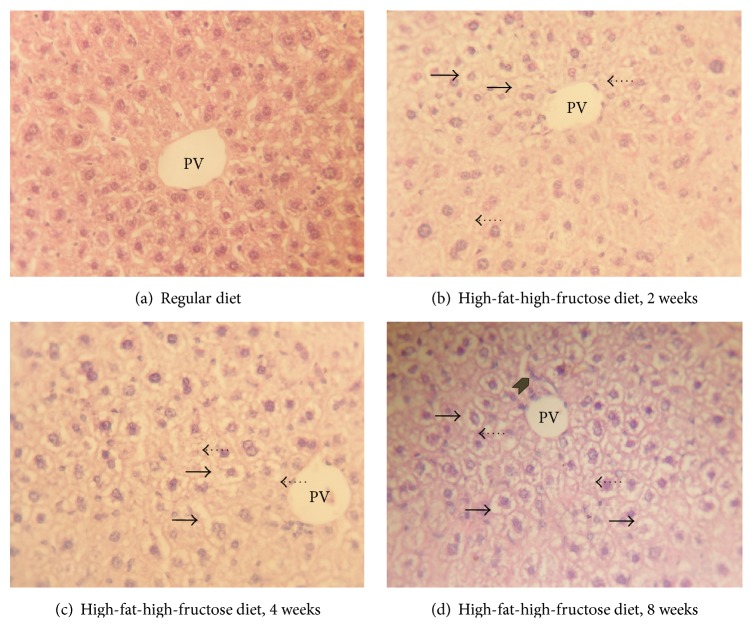
Histopathology of hematoxylin and eosin (H&E) stained mouse livers. The livers were collected 24 hours after the last treatment. Mice were fed a regular diet (a), or a high-fat, high-fructose diet for 2 weeks (b), 4 weeks (c), or 8 weeks (d). The micrographs are shown at a magnification of 400-fold. The solid arrows indicate sinusoidal pathology, the dash arrows indicate fat droplets, and the gray arrow indicates the foci of lobular inflammation in the liver. PV: portal vein.

Micrographs of H&E stained mouse hepatic tissues are shown in Figure 1. Compared with the mice fed the regular diet (Figure 1(a)), the liver sections of the mice fed the HFFD for 2 weeks exhibited steatosis as hepatocytes containing microvesicular fat droplets as clear areas (Figure 1(b)). After 4 weeks of the HFFD, the liver sections showed additional fat droplet accumulation and hepatocyte degeneration as sinusoidal pathology (Figure 1(c)). These pathological changes increased after 8 weeks of HFFD treatment with both microvesicular and macrovesicular fat droplets observed throughout the liver sections along with increased sinusoidal pathology and foci of inflammation (Figure 1(d)).

### 3.2. Antioxidant System Profile in the Livers of Mice Fed a High-Fat, High-Fructose Diet

**Figure 2:**
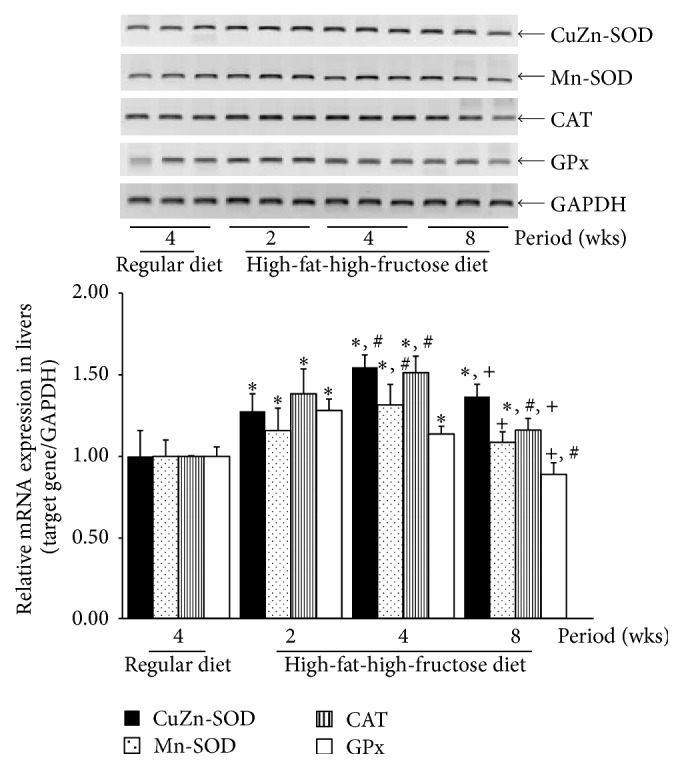
Effects of a high-fat, high-fructose diet on CuZn-SOD, Mn-SOD, CAT, and GPx mRNA expression in mouse livers. Mice were fed a commercial standard diet for 4 weeks, and a high-fat, high-fructose diet (HFFD) was administered intragastrically for 2, 4, or 8 weeks. A semiquantitative determination of the hepatic mRNA expression of certain genes was performed using pairs of primers specific to the investigated genes. The data are presented as the mean ± SD (*n* = 5). Significant differences were determined using a one-way analysis of variance (ANOVA) followed by Tukey's* post hoc* test. ^*∗*^ *p* < 0.05 versus the regular diet mice; ^#^ *p* < 0.05 versus 2-week HFFD mice; ^+^ *p* < 0.05 versus 4-week HFFD mice.

**Figure 3:**
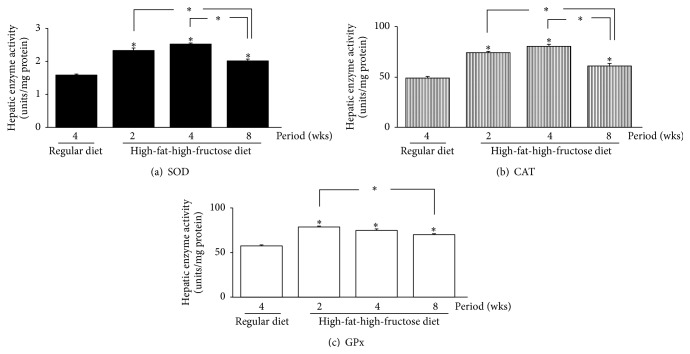
Effects of a high-fat, high-fructose diet on hepatic SOD, CAT, and GPx enzymatic activity. Mice were fed a commercial standard diet for 4 weeks, and a high-fat, high-fructose diet (HFFD) was administered intragastrically for 2, 4, or 8 weeks. The mice were sacrificed 24 h after the last treatment, and their livers were immediately excised to determine the activities of the SOD, CAT, and GPx enzymes. The data are presented as the mean ± SD (*n* = 5). Significant differences were determined using a one-way analysis of variance (ANOVA) followed by Tukey's* post hoc* test. ^*∗*^ *p* < 0.05 versus the regular diet mice.

**Figure 4:**
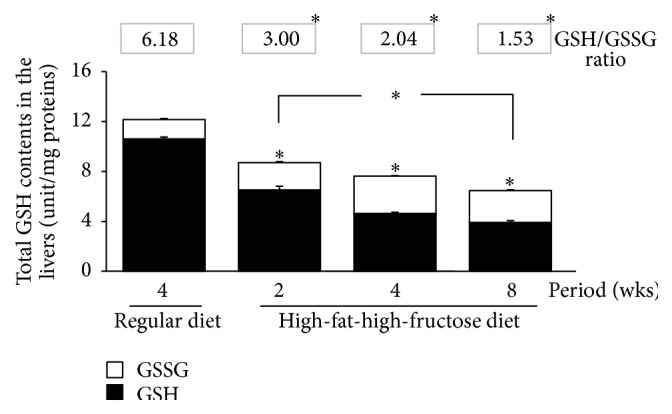
Effects of a high-fat, high-fructose diet on the total GSH, reduced GSH, and GSSH content and the GSH/GSSG ratio in the mouse livers. Mice were fed a commercial standard diet for 4 weeks, and a high-fat, high-fructose diet (HFFD) was administered intragastrically for 2, 4, or 8 weeks. The mice were sacrificed 24 h after the last treatment, and their livers were immediately excised to examine the total glutathione, reduced GSH (black column), and GSSG (white column) contents, and the GSH/GSSH ratio was calculated (digit above the graph). The data are presented as the mean ± SD (*n* = 5). Significant differences were determined using a one-way analysis of variance (ANOVA) followed by Tukey's* post hoc* test. ^*∗*^ *p* < 0.05 versus the regular diet mice.

**Figure 5:**
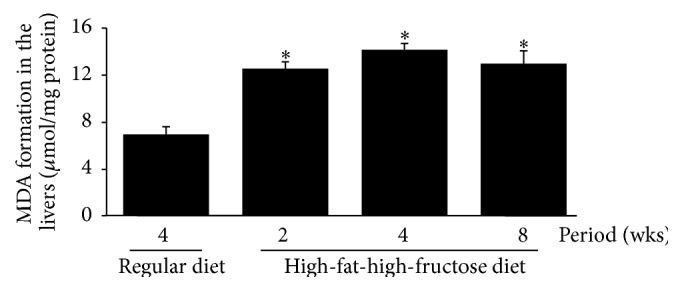
Effects of a high-fat, high-fructose diet on lipid peroxidation in the mouse livers. Mice were fed a commercial standard diet for 4 weeks, and a high-fat, high-fructose diet (HFFD) was administered intragastrically for 2, 4, or 8 weeks. The mice were sacrificed 24 h after the last treatment, and their livers were immediately excised to determine the levels of malondialdehyde (MDA). The data are presented as the mean ± SD (*n* = 5). Significant differences were determined using a one-way analysis of variance (ANOVA) followed by Tukey's* post hoc* test. ^*∗*^ *p* < 0.05 versus the regular diet mice.

The level of CuZn-SOD mRNA was significantly upregulated in the livers of mice fed the HFFD (Figure 2). The mRNA level of CuZn-SOD continually increased, with the highest expression after 4 weeks followed by a gradual decline at 8 weeks (Figure 2). The level of Mn-SOD mRNA was significantly increased in the HFFD-fed mouse livers. The level of CAT mRNA was similar to that of CuZn-SOD, while the level of GPx mRNA significantly increased for the first two weeks of the HFFD treatment but then started to decline at 4 weeks to nearly the same level as that of the normal mice at 8 weeks. Corresponding to the levels of mRNA expression (Figure 2), SOD, CAT, and GPx activities were significantly elevated in the mouse livers throughout the HFFD treatment for 8 weeks (Figures 3(a)–3(c)), though the enzymatic activities notably declined at 8 weeks. The total glutathione content and the GSH/GSSG ratio decreased sharply at all stages of the HFFD treatment whereas there was a significant increase in GSSG (Figure 4), indicating the depletion of the GSH stores. Moreover, the level of lipid peroxidation, presented as the concentration of MDA, was excessively raised in the HFFD mouse livers throughout the treatment period (Figure 5).

### 3.3. Antioxidant System Profile in the Brains of Mice Fed a High-Fat, High-Fructose Diet

**Figure 6:**
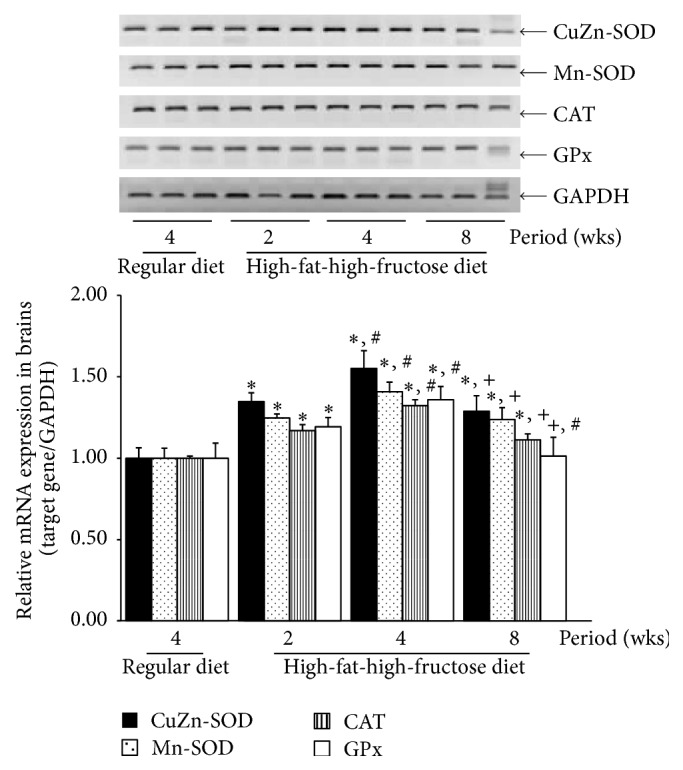
Effects of a high-fat, high-fructose diet on CuZn-SOD, Mn-SOD, CAT, and GPx mRNA expression in the mouse brains. Mice were fed a commercial standard diet for 4 weeks, and a high-fat, high-fructose diet (HFFD) was administered intragastrically for 2, 4, or 8 weeks. A semiquantitative determination of the mRNA expression of certain genes in mouse brain was performed using pairs of primers specific to the investigated genes. The data are presented as the mean ± SD (*n* = 5). Significant differences were determined using a one-way analysis of variance (ANOVA) followed by Tukey's* post hoc* test. ^*∗*^ *p* < 0.05 versus the regular diet mice; ^#^ *p* < 0.05 versus 2-week HFFD mice; ^+^ *p* < 0.05 versus 4-week HFFD mice.

**Figure 7:**
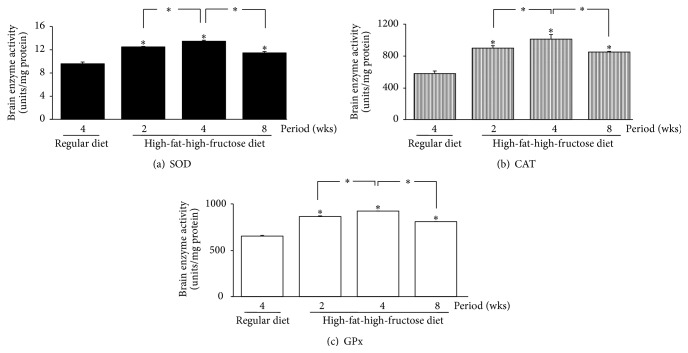
Effects of a high-fat, high-fructose diet on the SOD, CAT, and GPx enzymatic activity in the mouse brains. Mice were fed a commercial standard diet for 4 weeks, and a high-fat, high-fructose diet (HFFD) was administered intragastrically for 2, 4, or 8 weeks. The mice were sacrificed 24 h after the last treatment, and their brains were immediately excised to determine the SOD, CAT, and GPx enzyme activities. The data are presented as the mean ± SD (*n* = 5). Significant differences were determined using a one-way analysis of variance (ANOVA) followed by Tukey's* post hoc* test. ^*∗*^ *p* < 0.05 versus the regular diet mice.

**Figure 8:**
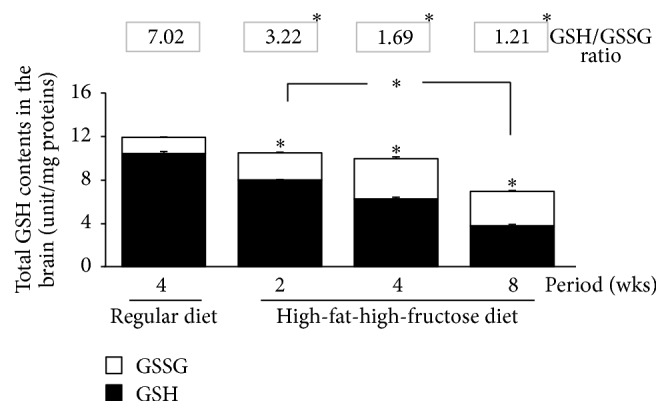
Effects of a high-fat, high-fructose diet on the total GSH, reduced GSH, and GSSH content and the GSH/GSSG ratio in the mouse brains. Mice were fed a commercial standard diet for 4 weeks, and a high-fat, high-fructose diet (HFFD) was administered intragastrically for 2, 4, or 8 weeks. The mice were sacrificed 24 h after the last treatment; their brains were immediately excised to determine the total glutathione, reduced GSH (black column), and GSSG (white column) content, and the GSH/GSSH ratio was then calculated (digit above the graph). The data are presented as the mean ± SD (*n* = 5). Significant differences were determined using a one-way analysis of variance (ANOVA) followed by Tukey's* post hoc* test. ^*∗*^ *p* < 0.05 versus the regular diet mice.

**Figure 9:**
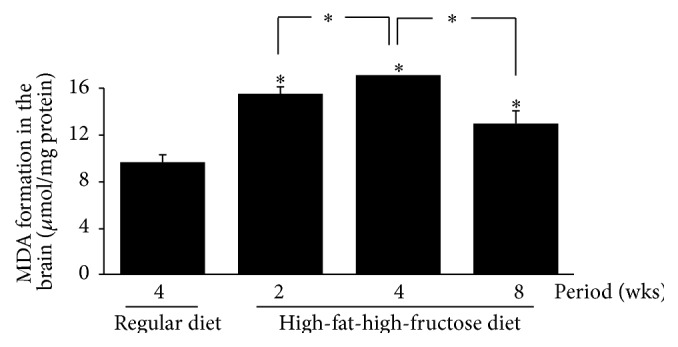
Effects of a high-fat, high-fructose diet on lipid peroxidation in the mouse brains. Mice were fed a commercial standard diet for 4 weeks, and a high-fat, high-fructose diet (HFFD) was administered intragastrically for 2, 4, or 8 weeks. The mice were sacrificed 24 h after the last treatment, and their brains were immediately excised to determine the levels of malondialdehyde (MDA). The data are presented as the mean ± SD (*n* = 5). Significant differences were determined using a one-way analysis of variance (ANOVA) followed by Tukey's* post hoc* test. ^*∗*^ *p* < 0.05 versus the regular diet mice.

The levels of all investigated antioxidant mRNAs, including CuZn-SOD, Mn-SOD, CAT, and GPx, were significantly increased in the HFFD mouse brains, with the highest levels at week 4, followed by a gradual decline at 8 weeks (Figure 6). The patterns of SOD, CAT, and GPx activities in the brains corresponded with the mRNA levels (Figure 7); the enzymatic activities gradually but significantly increased in the HFFD mouse brains and reached the maximal level at week 4 before dropping at week 8. The total glutathione content and the GSH/GSSG ratio were greatly reduced, with a significant increase in GSSG content throughout the duration of the HFFD treatment (Figure 8). Additionally, a significant increase in MDA production was observed in the HFFD mouse brains (Figure 9). These observations suggest that enhanced lipid peroxidation occurred in the HFFD mouse brains.

## 4. Discussion

Excess consumption of carbohydrates (fructose and sucrose) and fats (fatty acids and cholesterol) plays a key role in the development and progression of NAFLD through the activation of high-fat diet-modulated lipid metabolic pathways [13, 20]. Excessive carbohydrate and/or fat intake has also been shown to increase the concentrations of both blood glucose and free fatty acids, contributing to the accumulation of neutral lipids in the liver [21]. The hepatic metabolites of fructose, namely, fructose-1-phosphate and fructose-1,6-bisphosphate, further increase the storage of free fatty acids (FFA) in the liver and can cause significantly higher adenosine triphosphate (ATP) degradation and uric acid production in cirrhotic patients [22]. The lipogenesis of FFA in the liver subsequently causes impaired glycogen synthesis and insulin resistance in hepatocytes [23, 24] and adipocytokine release causes hepatocyte lipotoxicity, which damages hepatocytes through induction of apoptosis and recruitment of proinflammatory mediators [25]. In the present study, we fed mice a high-fat, high-fructose diet (HFFD) which has been shown to cause overnutrition in ICR mice [26, 27]. The energy from fat and fructose in the HFFD was 49.6% and 28.0%, of the total energy given and additional energy intake of 9.2 kcal/day. Lieber et al. [28] reported a dietary model of NASH that used a high fat diet (71% of energy from fat) in rats. Rats fed the high-fat diet for 3 weeks showed insulin resistance, marked panlobular steatosis, and hepatic lipid accumulation. In addition, hepatic oxidative damage, steatosis, and inflammation were noted in the rats fed high-fat diet, and these abnormal symptoms were reduced using a restricted dietary consumption program [29]. This corresponds to our results in mice fed the HFFD. Microvesicular fat droplets were observed after 2 weeks of HFFD treatment, and inflammatory histopathology was found in the mouse livers at 8 weeks.

The progression of nonalcoholic steatosis to steatohepatitis has been linked to the action of reactive oxidant species (ROS) in the liver [30, 31]. ROS are generated in the liver through several pathways, such as the mitochondria, peroxisomes, cytochrome P-450, reduced nicotinamide adenine dinucleotide oxidase, cyclooxygenase, and lipoxygenase. In both NASH and experimental steatohepatitis, the expression of hepatic CYP2E1 increased, leading to oxidative stress [32]. Oxidative stress can damage proteins or unsaturated lipids in cell membrane [33]. Here we report that MDA levels were elevated in the liver and brain of mice fed the HFFD, indicating that lipid peroxidation increased in the HFFD-fed mice presumably due to oxidative stress. We then examined the antioxidant systems through SOD, CAT, and GPx mRNA expression levels and enzymatic activity, together with the nonenzymatic antioxidant glutathione system [34, 35]. Dhuley reported the elevation of SOD, CAT, and GPx, in the livers and hearts of rats receiving a diet supplemented with 20% coconut oil for 90 days as well as increased MDA and hydroperoxide levels along with a decrease in glutathione content [36]. A study comparing oxidative stress in chronic hepatitis patients with NAFLD patients showed that the MDA level and the GPx and SOD activities were elevated, while the CAT activity and the glutathione content were suppressed in the plasma of the NAFLD patients [34]. Here, we present evidence that the mRNA expression and enzymatic activity of SOD, CAT, and GPx were increased and the total glutathione, the reduced GSH, and the GSH/GSSG ratio were decreased in both livers and brains of mice fed the HFFD for 2–8 weeks.

The cytosolic (CuZn-SOD) and mitochondrial (Mn-SOD) SOD enzymes catalyze the dismutation of superoxide (O_2_^−^) into oxygen (O_2_) and hydrogen peroxide (H_2_O_2_) [37]. Together, the SOD enzymes keep the intracellular steady-state concentration of (O_2_^−^) low, while CAT and GPx remove the H_2_O_2_ generated from the SOD reaction. GPx uses GSH to catalyze the transformation of H_2_O_2_ (or ROOH) to H_2_O (or ROH) and GSSG [38]. In the livers and brains of the HFFD-fed mice in the present study, GPx activity and GSSG content increased, while GSH content decreased, reducing the GSH/GSSG ratio. These findings indicate that the HFFD caused oxidative stress due to increased production of superoxide (SOD) and hydrogen peroxide (CAT and GPx).

## 5. Conclusion

The continuous consumption of a high-fat, high-fructose diet caused oxidative stress in mice: SOD, CAT, and GPx enzyme activities increased, while GSH stores were depleted. This manifested as histopathological damage to the liver including steatosis, inflammation, and damage to hepatocytes. Thus, the oxidant-antioxidant imbalance induced by long-term consumption of a high-fat, high-fructose diet appears to contribute to the development of NAFLD and increase the risk of progression to NASH.

## Figures and Tables

**Table 1:** Forward and reverse primers for mouse CAT, CuZn-SOD, Mn-SOD, GPx, and GAPDH.

Gene	Forward primer	Reverse primer	References
*CAT*	5′-GCA GAT ACC TGT GAA CTG TC-3′	5′-GTA GAAT GTC CGC ACC TGA G-3′	[17]
*CuZn-SOD*	5′-AAG GCC GTG TGC GTG CTG AA-3′	5′-CAG GTC TCC AAC ATG CCT CT-3′	[18]
*Mn-SOD*	5′-GCA CAT TAA CGC GCA GAT CA-3′	5′-AGC CTC CAG CAA CTC TCC TT-3′	[18]
*GPx*	5′-CCT CAA GTA CGT CCG ACC TG-3′	5′-CAA TGT CGT TGC GGC ACA CC-3′	[18]
*GAPDH*	5′-TCC ACT CAC GGC AAA TTC AAC G-3′	5′-TAG ACT CCA CGA CAT ACT CAG C-3′	[19]

**Table 2:** PCR programs for mouse CAT, CuZn-SOD, Mn-SOD, GPx, and GAPDH.

Gene	Denaturation (°C/sec)	Annealing (°C/sec)	Extension (°C/sec)	Number of cycles	Product size (bp)	References
*CAT*	94/45	55/60	72/60	31	229	[17]
*CuZn-SOD*	94/45	56/60	72/60	26	246	[18]
*Mn-SOD*	94/45	55/60	72/60	35	241	[18]
*GPx*	94/45	56/60	72/60	28	197	[18]
*GAPDH*	95/30	64/30	72/60	28	145	[19]

## Abbreviations

CAT: Catalase
CuZn-SOD: Copper-zinc superoxide dismutase
GPx: Glutathione peroxidase
GSH: Glutathione reduced form
GSSG: Glutathione oxidized form
HFFD: High-fat, high-fructose diet
Mn-SOD: Manganese superoxide dismutase
NAFLD: Nonalcoholic fatty liver disease
NASH: Nonalcoholic steatohepatitis
ROS: Reactive oxygen species
SOD: Superoxide dismutase.
